# Reverse Engineering of Radical Polymerizations by Multi-Objective Optimization

**DOI:** 10.3390/polym16070945

**Published:** 2024-03-29

**Authors:** Jelena Fiosina, Philipp Sievers, Gavaskar Kanagaraj, Marco Drache, Sabine Beuermann

**Affiliations:** 1 Institute of Informatics, Clausthal University of Technology, Julius-Albert-Str. 4, 38678 Clausthal-Zellerfeld, Germany; 2 Institute of Technical Chemistry, Clausthal University of Technology, Arnold-Sommerfeld-Strasse 4, 38678 Clausthal-Zellerfeld, Germany; philipp.sievers@tu-clausthal.de (P.S.); marco.drache@tu-clausthal.de (M.D.)

**Keywords:** polymerization reverse engineering, clustering, multi-objective optimization

## Abstract

Reverse engineering is applied to identify optimum polymerization conditions for the synthesis of polymers with pre-defined properties. The proposed approach uses multi-objective optimization (MOO) and provides multiple candidate polymerization procedures to achieve the targeted polymer property. The objectives for optimization include the maximal similarity of molar mass distributions (MMDs) compared to the target MMDs, a minimal reaction time, and maximal monomer conversion. The method is tested for vinyl acetate radical polymerizations and can be adopted to other monomers. The data for the optimization procedure are generated by an in-house-developed kinetic Monte-Carlo (kMC) simulator for a selected recipe search space. The proposed reverse engineering algorithm comprises several steps: kMC simulations for the selected recipe search space to derive initial data, performing MOO for a targeted MMD, and the identification of the Pareto optimal space. The last step uses a weighted sum optimization function to calculate the weighted score of each candidate polymerization condition. To decrease the execution time, clustering of the search space based on MMDs is applied. The performance of the proposed approach is tested for various target MMDs. The suggested MOO-based reverse engineering provides multiple recipe candidates depending on competing objectives.

## 1. Introduction

Radical polymerizations are known to be very robust and to provide access to a wide range of polymers with largely differing properties, which are defined by the process conditions. The strong correlation between the production process and material properties is due to the complex reaction mechanism consisting of a large number of elemental reactions, even for homopolymerizations with a single monomer [1]. The kinetics of the elemental reactions are strongly dependent on the process conditions. Therefore, the prediction of a suitable radical polymerization process to obtain a polymer with targeted properties is challenging. To allow for on-demand polymer synthesis, at first sight, it appears highly attractive to apply simulations of polymerization processes, e.g., employing differential equations [2,3,4] or kinetic Monte-Carlo (kMC) methods [5,6,7,8]. Simulations are particularly valuable, because detailed information on polymer microstructure at each time moment is available, which is not accessible from polymerization processes. However, this type of simulation cannot be run backwards, and the on-demand suggestion of polymerization conditions to obtain a pre-defined polymer is not feasible. To overcome this issue, reverse engineering has the potential to provide several solutions, as opposed to a single one-to-one relation between polymerization variables and microstructural properties [9]. From the input variables, a polymerization process model predicts the concentration vs. time profiles and the polymer properties. Inverse modeling, on the other hand, is more difficult and calls for optimization strategies. In order to determine the optimal input values for systems with complex reaction mechanisms that provide pre-defined reaction outputs (such as pre-set conversion, yield, and/or other product properties), it was suggested to intelligently explore the reaction condition search space [10]. Further, it was proposed to solve reverse engineering problems using machine learning (ML)-based prediction [11], in which ML regression models based on the random forest algorithm and a multivariate and multi-target regression problem [12] were applied. The model took a targeted MMD and predicted the initial polymerization conditions to produce a polymer with this targeted property, minimizing the errors in predicted recipes. However, the prediction of the recipe was based on the MMD only and was not optimized with respect to multiple objectives like reaction time or conversion. 

Pareto or multi-objective optimization (MOO) [13] is the process of maximizing or minimizing many objective functions while taking a set of constraints into consideration. Numerous scientific domains, such as engineering [14], economics [15], and logistics [16], need the use of MOO when making optimum decisions facing trade-offs between two or more competing objectives. The increasing use of MOO has been seen in chemical engineering [17,18]. In 2009, Fiandaca et al. [19] used genetic algorithm-based MOO to optimize a pressure swing adsorption process based on the maximization of two objectives: nitrogen recovery and nitrogen purity. In 2013, Ganesan et al. [20] carried out MOO of combined carbon dioxide reforming and partial oxidation of methane with respect to three objectives. MOO utilized a gravitational search algorithm and particle swarm optimization to tackle the problem. With respect to technical applications, it appears highly important to solve the reverse engineering problem as an optimization task with multiple contradicting objectives [13]. Various ML-based optimization strategies were addressed for the purpose of reverse engineering polymerization processes [21,22,23,24,25]. A genetic algorithm-based optimizer was proposed by Mohammadi et al. [9] to generate a variety of polymerization recipes at random and to send them to the kMC simulator for error evaluation. 

**Figure 1:**
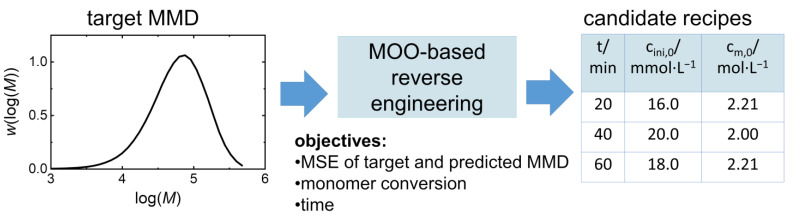
Illustration of the polymerization reverse engineering approach by means of MOO.

This study provides a MOO-based reverse engineering approach, not only ensuring that the targeted MMD is obtained by means of minimizing the mean squared error (MSE), but also providing a minimal reaction time and maximal monomer conversion. Frequently, there are several recipes for obtaining similar MMDs, which are referred to as candidate recipes in the following discussion. The solution of the MOO approach is referred to as a polymerization recipe, which includes temperature, reaction time, as well as initial monomer and initiator concentrations. In the proposed MOO approach, the data for the selection of the optimal recipes from the search space are based on kMC simulations, as previously reported for training ML models [11]. The simulation provides the dependence of monomer conversion and the corresponding MMD on the polymerization conditions and reaction time. The proposed reverse engineering algorithm consists of several steps. First, kMC simulations are run for the selected recipe search space to derive the MMDs and monomer concentrations as an input for the MOO step. Then, MOO is applied for a given target MMD, and the Pareto optimal space is found on the base of the search space. In the last step, a weighted sum optimization function is used to calculate the weighted score of each candidate recipe, which is used for evaluating the solutions. The best candidate has the smallest score. To accelerate the MOO procedure, additional search space clustering on the basis of MMDs is considered. The approach pursued is illustrated in Figure 1. The method was tested for the following model system: a chemically initiated vinyl acetate (VAc) radical polymerization model system at 60 °C using tert-butyl peroxypivalate as the initiator. This model system was chosen as an example, because of its industrial relevance. Poly(vinyl acetate) is used as a precursor of poly(vinyl alcohol) [26], which is widely used as a protective colloid in suspension and emulsion polymerizations [27].

## 2. Reverse Engineering Modeling Approach

### 2.1. Model Development

This section provides a formal description of solving a reverse engineering problem with a MOO approach. State-of-the-art MOO methods [28] like genetic algorithms require the generation and evaluation of new recipes in each step. Due to the high number of required steps, it is very demanding to perform on-line kMC simulations in loops [9]. 

Instead, here, a recipe search space **R** is selected, where each recipe **r** ∈ **R** consists of reaction time *t*, the initial monomer concentration *c*_m,0_, and the initial initiator concentration *c*_ini,0_. Then, the corresponding monomer concentration *c*_m_(**r**) and molar mass distribution *MMD*(r) are obtained for each **r** ∈ **R** via kMC simulation. In the MOO approach, the input is a target MMD, *MMD*^target^, and the output is a set of optimal candidate recipes R^*^: $r_{1}^{*}$,$r_{2}^{*}$, $\ldots ,r_{n}^{*}$. r^*^ is a subset of the recipe search space **R**, R^*^ ⊂ **R**, with MMD($r_{i}^{*}$) being close to *MMD*^target^, as evaluated on the basis of the MSE as well as the maximal conversion and minimal time. 

The optimization variables are presented in Table 1. The lower and upper limits for the variables *c*_m,0_, *c*_ini,0_, and *t* are defined by the simulated data. In Table 2, for a specific recipe **r**, the simulated values *c*_m_(**r**) and *MMD*(**r**) are used to calculate the values of three optimization objective functions: objective for the reaction time, *f*_t_(**r**), and objective for the mean squared error (MSE) between *MMD*^target^ and the predicted *MMD*(**r**), *f*_MSE_. *f*_cm_(**r**) is used to turn the maximization problem of monomer conversion *f*_conv_(**r**) = (*c*_m,0_ − *c*_m_(**r**))/*c*_m,0_ into a minimization problem, in which 1 − *f*_conv_(**r**) is minimized (Table 2).

The final decision takes user preferences into account by assigning specific weights to the objectives applying the weighted sum method [28,29]. Thus, the multi-objective function can be represented in a single-objective way. Then, the values of this function are calculated for each candidate recipe, and the recipes with minimal values of the objective function are selected as a set of optimal solutions. A weight *w_i_* is assigned to each normalized objective function *f_i_* as follows:

(1) $$\underset{r}{\min}f\left(r\right)=\underset{r}{\min}\sum_{i}w_{i}f_{i}\left(r\right)$$

where $\sum_{i}w_{i}=1$*, i* ∈ {MSE, cm, *t*}, **r** ∈ **R,** and **R** is a polymerization recipe space. For clarity of presentation, we avoid the double indexing and use “cm” instead of “*c*_m_“ when it is used as a subscript. Additionally, the weight *w*_cm_ of the objective *f*_cm_ is also referred to as the weight of the conversion objective. The MOO model is universal for solving reverse engineering problems for other polymerizations. The number of objectives and their description can be customized, e.g., the conversion of initiator can be added as an objective.

**Figure 2:**
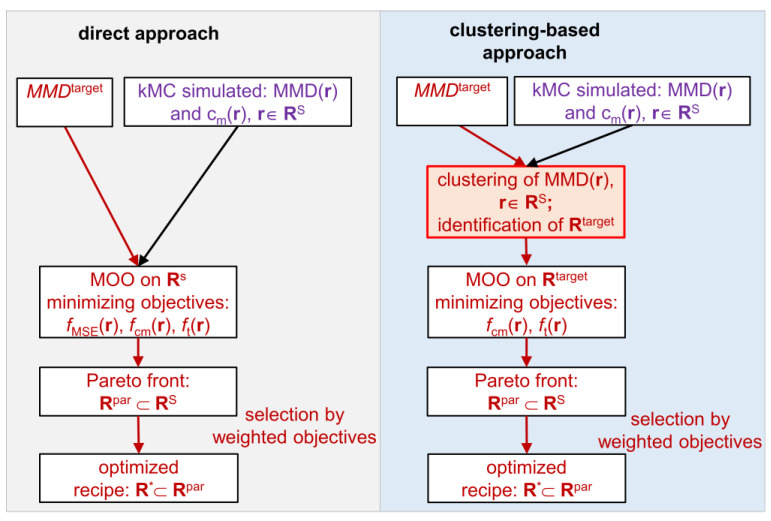
Direct Pareto optimization vs. clustering-supported Pareto optimization.

The steps of the proposed algorithm are presented in Figure 2 as a direct approach. First, a search space **R**^S^ ⊂ **R** is selected (for details, see Section 2.2), and for each **r** ∈ **R**^S^, MMD(**r**) and *c*_m_(**r**) are obtained via kMC simulations. Then, MOO is performed over **R**^S^. First, the objective function values are calculated as follows: the values for the objective *f*_t_(**r**) are already included in **r** as *t*, the values of the objective *f*_cm_(**r**) are calculated in advance for all possible **r** according to Table 2, and the values of the objective *f*_MSE_ are specified by *MMD*^target^. Further, based on the calculated objective function values, the Pareto front points **R**^par^ ⊂ **R**^S^ leading to *MMD*^target^ are identified. For this, the points from **R**^S^ are shown in the Pareto optimal space, with coordinates specified by the three objectives. The Pareto front points are found in this Pareto optimal space, such that one value of the objective function cannot be improved without downgrading the value of another objective function [13]. Finally, for the Pareto front points **R**^par^, the weights of each objective function are defined, and a set of the best recipe candidates R^*^ ⊂ **R**^par^ according to Equation (1) is selected.

**Figure 3:**
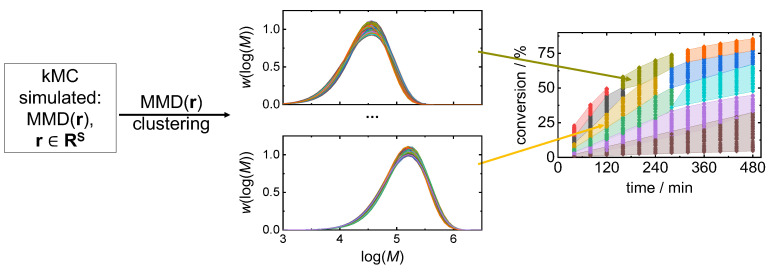
Illustration of the clustering approach of the search space. In the middle two graphs, exemplary clusters of MMDs are presented, on the right, each MMD cluster defined is represented by a different color.

The above-described algorithm is improved with respect to the optimization time by means of clustering the search space, which is illustrated in Figure 2 as the clustering-supported approach. Clustering divides the search space **R**^S^ into a number of clusters, as illustrated in Figure 3. First, the search space **R**^S^ is clustered on the base of MMD(**r**), which allows for the selection of a cluster **R**^target^ ⊂ **R**^S^ containing the MMDs, which are the closest to *MMD*^target^. In general, a larger number of clusters leads to a smaller number of MMDs per cluster gaining higher similarity with the distributions. However, there is less space for optimization regarding other objectives, e.g., such as polymerization time and monomer conversion. For this reason, an appropriate trade-off between the number of clusters and their size has to be identified. Upon appropriate clustering, a cluster for the target MMD (Figure 2, red arrows) **R**^target^ is found. Then, by MOO, the search space is reduced to the number of Pareto front points **R**^par^ ⊂ **R**^target^. Finally, after defining objective weights, the best recipe candidates R^*^ ⊂ **R**^par^ are found according to Equation (1). Since MOO is applied to a single cluster **R**^target^ ⊂ **R**^S^, which is considerably smaller than **R**^S^, the optimization time is significantly reduced.

Different methods can be applied for clustering the search space on the basis of MMD. The clustering of distributions and their representation in histograms is an important topic which attracted a lot of attention because of specific metrics which should be used to compare distributions. One of the most popular and fast clustering methods is the kMeans method [30]. A modified kMeans clustering algorithm was applied to the clustering of histograms [31]. Further, a novel non-parametric clustering algorithm of empirical probability distributions was proposed [32]. Here, the classical kMeans clustering method was used. This algorithm starts with a random separation of the MMDs into clusters. At each step, it recalculates the centroids of each cluster and relocates the data points to the new centroids. The clustering process finishes when the clusters are stable or the given number of iterations is reached. In this study, the simplest Euclidean distances are used for the calculation of the distances between multi-dimensional data points, while specific metrics for the clustering of distributions are also available [31,33]. 

There are different strategies for data generation for the MOO procedure: the use of data exclusively generated in advance from kMC simulations, on-demand kMC-generated data, ML-generated data, kMC-based and ML-generated hybrid data sets, etc. Currently, as a first step, the focus is exclusively on the use of kMC-simulated data. 

### 2.2. Data Acquisition and Processing

The in-house-developed kMC simulator, mcPolymer, was used to carry out the simulations required for the generation of polymerization data according to the search space [34]. This simulator allows for the exportation of the concentration profiles of all reactants and products as well as microstructural data like the MMD, chain composition, and branching of all polymeric species involved in the process. The simulator output was adapted to be easily machine readable. The data were filtered, further abstracted, logically connected, and stored in the well-structured no-SQL database, MongoDB. The kMC simulated MMDs and monomer concentrations were obtained for the selected search space **R**^S^, allowing for MOO and for the weighted optimal solution to subsequently be found. 

The kMC simulations were performed for radical polymerizations with VAc as the monomer, tert. butyl peroxypivalate as the initiator, and methanol as the solvent. The simulations were based on a full kinetic model for VAc radical polymerization containing all elemental reactions. Previously, it was shown that the kinetic model used describes a large number of experimental data very well [35]. The following polymerization conditions were used: constant temperature of 60 °C, *c*_ini,0_ in the range of 1.0 to 20.0 mmol·L^−1^, and *c*_m,0_ in the range of 2.0 to 5.0 mol·L^−1^ with a uniformly distributed grid size of *c*_ini,0_ (geometrically scaled grid points) and *c*_m,0_ (arithmetic scaled grid points), resulting in 225 simulations of the process. The geometric scale was selected for *c*_ini,0_ to put more attention on the small values of this parameter. The application of a uniformly distributed grid and the random selection of the test data ensured the good balance of the datasets used for training and testing. The polymerization process was simulated for a constant reaction time of 6 h, and the properties of interest were recorded every 20 min, thus obtaining 18 data points in total at different time moments for each investigated property. Thus, the data set contained 4050 different MMDs, which were selected in a way that sufficiently covered the relevant technical reaction conditions. The number of data is reasonable in view of the simulation time. The simulations took 9 h with 128 CPU cores (2 AMD EPYC 7H12) and 2 TB of RAM. Moreover, this number of 4050 MMDs allowed for the construction of machine learning models for reverse engineering and MMD prediction with good performance [11].

For training the ML prediction models for single-objective reverse engineering, the obtained data set was divided into a training and test set in the proportion of 80:20. The same data were used for MOO, again by randomly taking 80% of the data as the training set for the search space **R**^S^ and 20% of the data as the test set **R**^test^. The test set **R**^test^ contained 810 recipes, which corresponded to a set **MMD**^test^ consisting of 810 kMC-simulated MMDs. Such a big test set allowed us to test the sensitivity of the proposed approach to the quality of input data. The evaluation of all optimization approaches was performed with **MMD**^test^, with each element serving as *MMD*^target^. In order to test the MMO approach, a single MMD was selected from **MMD**^test^ and used as *MMD*^target^ for the MMO approach. The performance of the MMO was evaluated by testing it with every MMD from **MMD**^test^.

## 3. Results and Discussion

**Figure 4:**
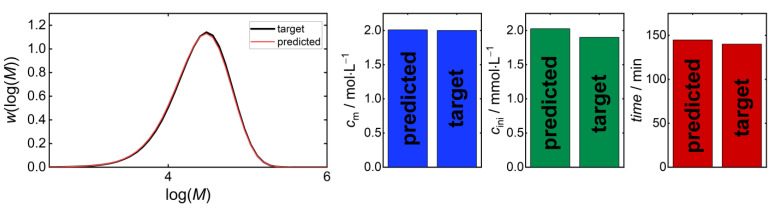
An example of reverse engineering with ML modeling comparing the MMD (**left**) and the recipe (**right**) for a single *MMD*^target^, with *MMD*^target^ being an element of **MMD**^test^. The target and predicted MMDs are overlapping, because their MSE is very small and equals 7.26 × 10^−5^. The target recipe is the recipe associated with *MMD*^target^.

In order to compare results obtained via the previously described ML modeling-based reverse engineering strategy [11] with data from the MOO approach introduced in this work, the technique reported for butyl acrylate was adopted for the polymerization of vinyl acetate. Previously, it was described how an ML regression model provides a recipe **r** = (*c*_m,0_, *c*_ini,0_*, T*) for a fixed time for a given *MMD*^target^. *c*_m,0_ and *c*_ini,0_ are initial concentrations of the monomer and the initiator, respectively, and *T* is the polymerization temperature. The prediction was performed with the random forest method for a target MMD to minimize the errors in the predicted recipe **r**(MMD). In the case of VAc polymerizations, the temperature was kept constant at *T* = 60 °C, and the reaction time *t* was predicted by the following model: **r** = (*c*_m,0_, *c*_ini,0_*, t*). The reverse engineering result for a sample *MMD*^target^ presented in Figure 4 corresponds to the recipe (*c*_m,0_; *c*_ini,0_; *t*)/(2.0 mol/L; 1.9 mmol/L; 140 min). Only very small differences between the target and predicted MMD as well as the predicted recipe were seen. The overall performance of the developed model was evaluated using the *R*^2^ determination coefficient, which is a measure for the quality of the prediction by the ML model. Keeping in mind that *R*^2^ = 1 indicates perfect prediction, it is remarkable to note that a rather low value of 0.78 for *R*^2^ is obtained, while the visual inspection of the MMDs for the representative example given in Figure 4 indicates only minor differences. The target and predicted MMDs are overlapping because their MSE is very small and equals 7.26 × 10^−5^. The MSEs of all target MMDs from the test set are presented in Figure 7. However, this ML-based approach does not consider multiple contradicting optimization objectives and does not provide multiple alternative solutions as proposed in the Pareto optimization. 

### 3.1. Direct Pareto Optimization

**Figure 5:**
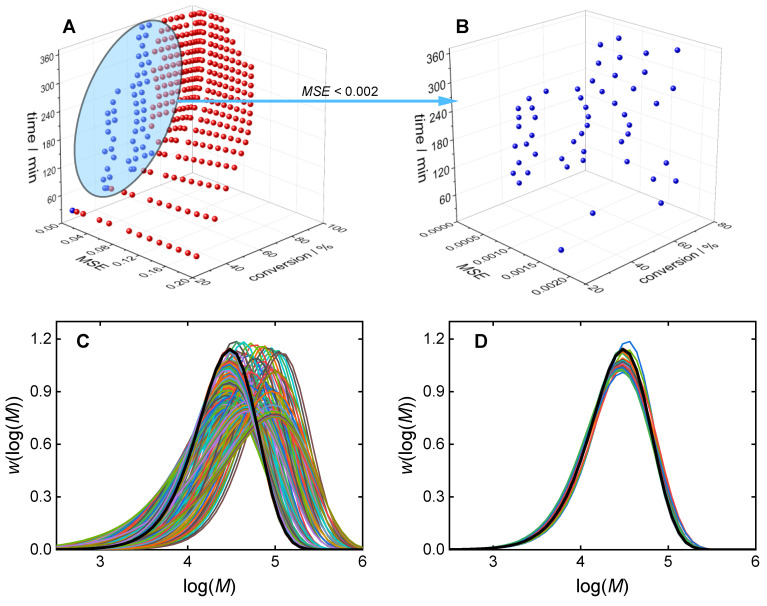
Pareto front points (**A**), filtered Pareto front points (**B**), MMDs for all Pareto front points (**C**), and MMDs for filtered Pareto front points (**D**). The target MMD is shown in a bold black line; the MSE limit was set to 2 × 10^−3^.

Figure 5A shows all Pareto front points with three objectives *f_i_*, *i* ∈ {MSE, cm, *t*}. After the definition of objective weights, the optimal solution from the Pareto front points was selected, which satisfied the weighted score calculated with Equation (1) best. Several combinations of objective weights were considered: time focused, conversion focused, and MSE focused. For example, in the time-focused case, the weight of time was higher than the other weights. To identify the optimal recipe leading to the target MMD, the MSE should have a higher weight than the other objectives. To reduce the number of Pareto front points, an MSE limit was chosen. As an example, the data in Figure 3 were obtained with an MSE limit of 2×10^−3^.

**Figure 6:**
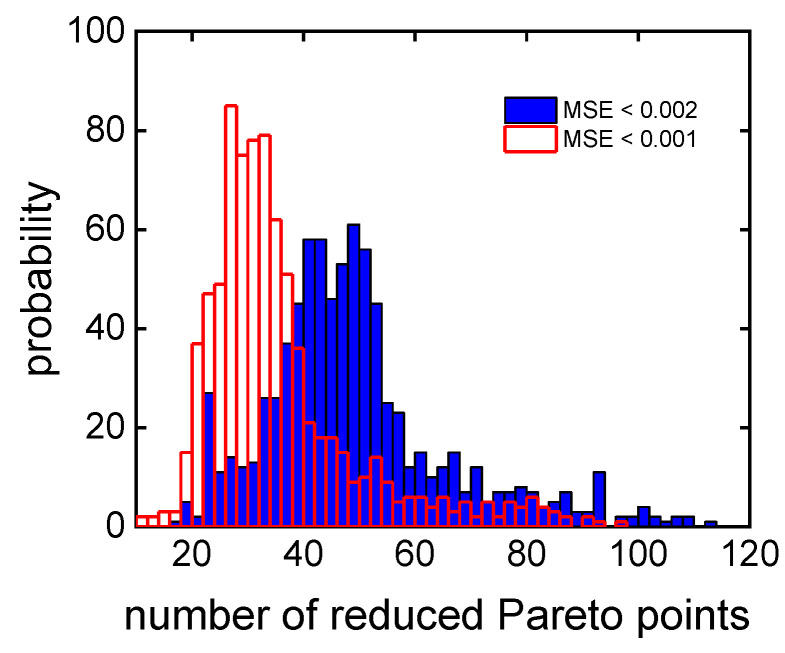
The influence of the MSE limit on the distribution of the number of filtered Pareto front points based on **MMD**^test^.

The optimization procedure is illustrated in Figure 5 for an example target MMD, which corresponds to the recipe (*c*_m,0_; *c*_ini,0_; t)/(2.0 mol/L; 1.9 mmol/L; 140 min). Rather than presenting the objective *f*_cm_, which represents the minimized monomer concentration, the conversion is shown in Figure 5A,B. Figure 5B demonstrates the reduction in the number of Pareto front points from 340 points (A) to 46 points (B) by filtering with the MSE limit of 2 × 10^−3^. The MMDs of all Pareto front points and of the filtered Pareto front points are given in Figure 5C,D, respectively. The filtering approach allows for the consideration of only MMDs of similar shape. Figure 6 shows the impact of different MSE limits on the number of Pareto front points. Even with an MSE limit of 10^−3^, the average number of points is around 30, which is a relevant number for the consideration of other objectives. The minimal number of filtered Pareto points for the MSE limit of 10^−3^ is about 10.

**Figure 7:**
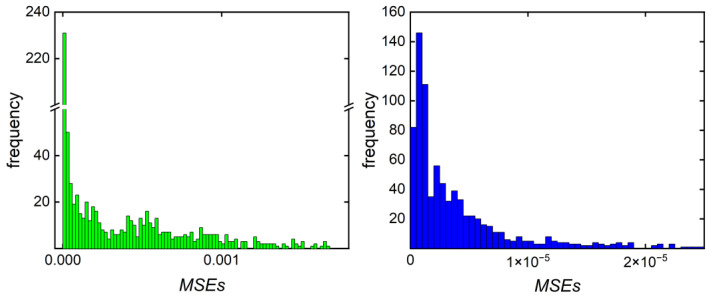
MSEs of the MMDs of the recipes obtained by ML prediction (**left**) and MOO (**right**) approaches on the basis of **MMD^test^**.

Figure 7 allows for the comparison of MSEs obtained via ML prediction-based (left) and MOO-based approaches (right) for reverse engineering calculated for all elements of **MMD**^test^. Minimal MSEs are preferable. For the ML-based prediction (left), the MSE values of almost all *MMD*^target^ from **MMD**^test^ are less than 10^−3^, while most MSEs obtained by the MOO-based approach with an MSE limit of 2 × 10^−3^ are by two orders of magnitude lower. Most values are less than 10^−5^. 

**Figure 8:**
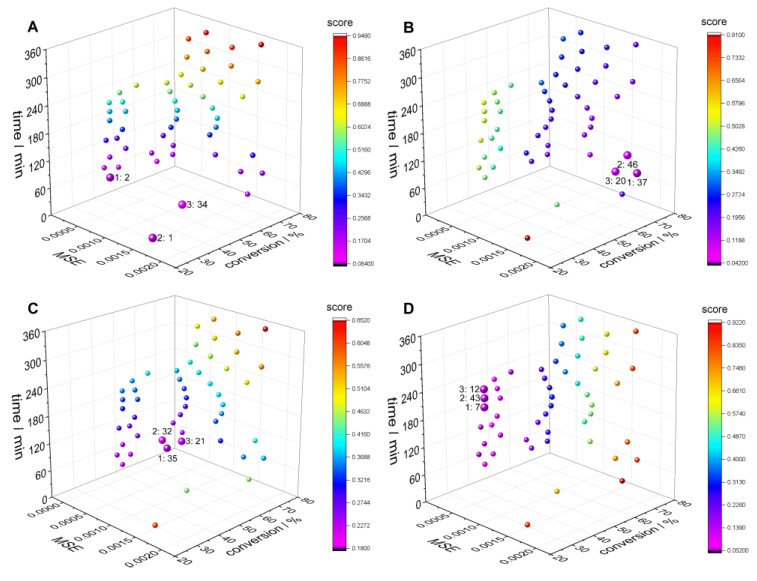
Influence of the combination of objective weights on the selection of the optimal recipes for a sample *MMD*^target^: time focused (*w*_MSE_ = 0.05, *w*_cm_ = 0.05, *w*_t_ = 0.9) (**A**), monomer conversion focused (*w*_MSE_ = 0.01, *w*_cm_ = 0.8, *w*_t_ = 0.19) (**B**), equal weights (*w*_MSE_ = 1/3, *w*_cm_ = 1/3, *w*_t_ = 1/3) (**C**), MSE focused (*w*_MSE_ = 0.9, *w*_cm_ = 0.09, *w*_t_ = 0.01) (**D**). The best three solutions are shown with their IDs for each case.

The next step is to evaluate the candidate recipes based on the weighted sum of the objective values according to Equation (1). Figure 8 and Table 3 present the candidate recipes for the different combinations of objective weights indicated. In Figure 8, a color code is assigned to the individual candidate recipes according to the score of the weighted sum of the values of the three objective functions *f*_MSE_, *f*_cm_, and *f*_t_ calculated according to Equation (1). For the weights *w*_MSE_ = 0.05, *w*_cm_ = 0.05, and *w*_t_ = 0.90, in Figure 8A, the focus is on the time. As a minimization problem is solved, a minimal score value (visualized by big pink points) determines the best candidates, given by the recipes with IDs 1, 34, and 2 in Table 3. Other weight combinations with focus on the monomer conversion in Figure 8B and with equal weights in Figure 8C demonstrate which recipe candidates are selected depending on specific requirements.

### 3.2. Clustering-Supported Pareto Optimization

**Figure 9:**
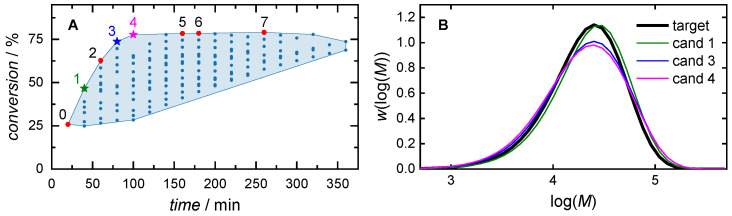
Clustering-supported Pareto optimization. (**A**) Illustration of the target cluster **R**^target^. The Pareto front points **R**^par^ ⊂ **R**^target^ are marked (colored markers) and are listed in Table 4. A time-focused result (*w*_cm_ = 0.2, *w*_t_ = 0.8), an equal-weighted result (*w*_cm_ = 0.5, *w*_t_ = 0.5) and a conversion-focused result (*w*_cm_ = 0.8, *w*_t_ = 0.2) are highlighted (1, 3, 4). The corresponding MMDs in comparison with *MMD*^target^ are shown in (**B**).

Clustering-supported optimization was performed for the same *MMD*^target^ as in the previous section. It was assumed that all points contained in the target cluster **R**^target^ had satisfactory MSEs. For this reason, only the two objectives of *f*_cm_ and *f*_t_ were chosen to be optimized. Figure 9 shows **R**^target^ in the coordinate space, which corresponds to the objective functions. The Pareto front points **R**^par^ ⊂ **R**^target^ are indicated by colored markers. Table 4 presents all Pareto front points with the corresponding recipes and objective weights.

To find the weighted solution for a time-focused result with *w*_cm_ *=* 0.2 and *w*_t_ = 0.8, the candidate with ID 1 reaching a conversion of 47% at a reaction time of 40 min is best. If conversion is considered to be more important than reaction time, as in the case of *w*_cm_ = 0.8 and *w*_t_ = 0.2, the candidate with ID 4 is the best solution, leading to a conversion of 77% with a reaction time of 100 min.

**Figure 10:**
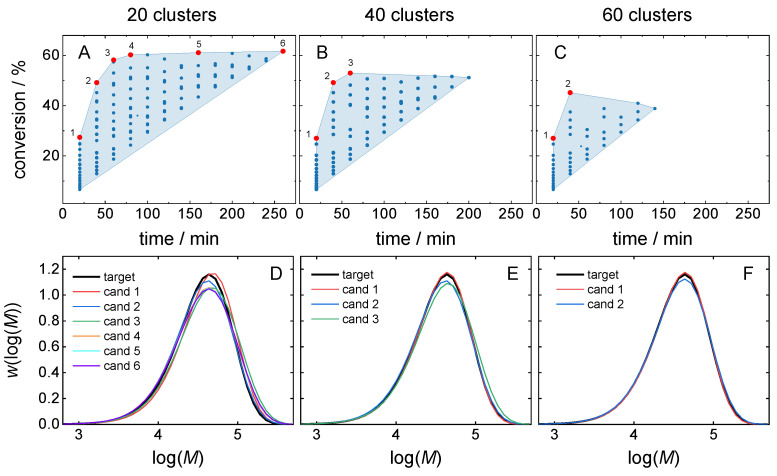
Cluster size dependence. (**A**–**C**) Clusters for *MMD*^target^ with a total number on 20 (**A**), 40 (**B**), or 60 (**C**) clusters. (**D**–**F**) MMDs for the Pareto front points.

MMD clustering focuses only on a single objective function *f*_MSE_ when identifying the most similar cluster for the target MMD. The accuracy is determined on the basis of the MSE, which represents the deviation of the selected solution from *MMD*^target^ and depends on the cluster size, and consequently, on the number of clusters. For a small number of clusters, every cluster contains a broader spectrum of MMDs as compared to a large number of clusters and leads to an increase in the MSEs of *MMD*^target^ and the MMDs contained in this cluster. However, the cluster covers a larger value range of the objective function, allowing for a broader range of predicted recipes. Figure 10 depicts how the *MMD^t^*^arget^ can be reached for clustering the search space into 20, 40, and 60 clusters, respectively. Figure 10A–C show the points of the target clusters in the objective function space. The shape of the clusters is similar. As the number of clusters grows, the number of candidates per cluster decreases, because there are fewer points fitting the required accuracy with respect to the MMD. Therefore, the size of the whole cluster shrinks. The red points in Figure 10 mark the Pareto front points, from which the optimal candidates are selected.

Figure 10D–F show the MMDs for all Pareto front points. With an increasing number of clusters, the MSE of *MMD*^target^ and Pareto front MMDs decreases. The objective function value range also decreases, as illustrated in Table 5. For example, the *f*_MSE_ of *MMD*^target^ improves by a factor of 100 when going from a total number of 20 clusters to a total number of 60 clusters. This improvement is achieved by a loss in conversion, which is reduced from 58% to 27%. In this example, the reaction time is also lowered with an increasing number of clusters. The last two rows in Table 5 provide the scores calculated by Equation (1) for the best recipes obtained with clustering of the search space into 20, 40, and 60 clusters for two cases with a different number of objectives. Note, that only the scores obtained with the same combination of weights (given in a single row of Table 4) are comparable. For equal weights of two objectives (*w*_cm_ = 0.5, *w*_t_ = 0.5), the minimal score of 0.39 was obtained with clustering into 40 clusters. Although the optimization was carried out within one cluster using only two objectives, the results can be related to the direct Pareto optimization, including the MSE objective. It is shown that for equal weights of three objectives (*w*_MSE_ = 1/3, *w*_cm_ = 1/3, *w*_t_ = 1/3), the minimal score of 0.30 was also obtained with the same clustering into 40 clusters. 

The comparison of the direct and the clustering-supported Pareto approach is based on the MSE limit. For the direct approach, the MSE limit can be chosen by the operator. For the clustering-supported approach, the individual MSE limit for a given *MMD*^target^, which evidently depends on the number of clusters, is defined as the maximal MSE value for all MMDs contained in **R**^target^.

**Figure 11:**
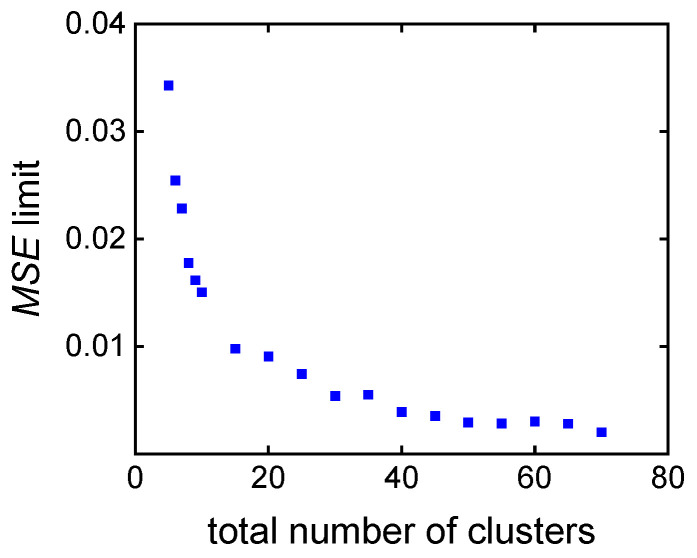
*MSE* limit calculated for the clustering of the search space.

Both approaches were compared on the basis of **MMD**^test^. Then, for the clustering-supported approach, the MSE limit was defined as the maximal value of all individual MSE limits over **MMD**^test^. Figure 11 shows the dependency of the MSE limit on the total number of clusters. For more than 30 clusters, the MSE limit is almost constant at a level of 0.003. The MSE limit increases rapidly for less than 30 clusters. 

**Figure 12:**
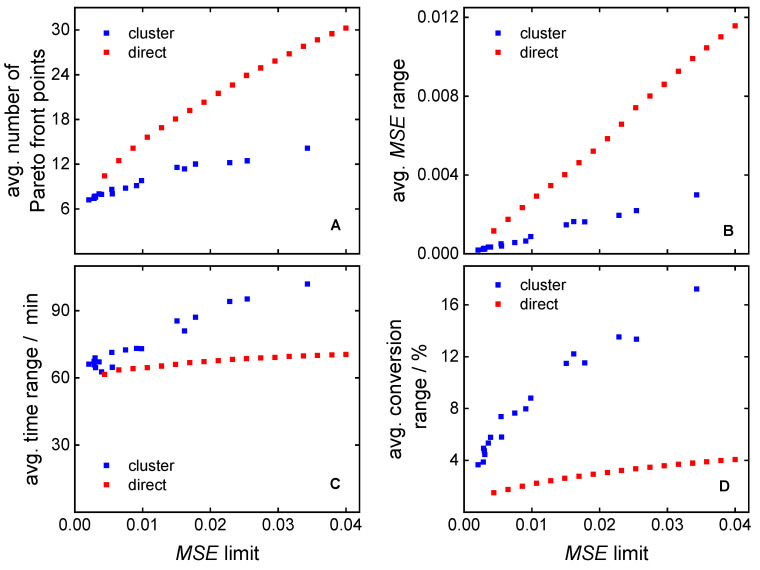
Comparison of the direct and the clustering-supported approach considering (**A**) the average number of Pareto front points, (**B**) the average MSE range, (**C**) the average polymerization time range, and (**D**) the average conversion range.

Figure 12A illustrates the average number of Pareto front points depending on the MSE limit. Increasing the MSE limit results in a reduction in the number of clusters (see Figure 11) associated with an enlargement in cluster size, and thereby leading to a higher count of Pareto front points. The clustering-supported approach is more selective considering the average number of Pareto front points. The standard deviation was used to describe the value ranges of the objective function and of the MSE function. Wider ranges are advantageous, because they provide more space for optimization. Averaging the value ranges of each objective function over **MMD**^test^ yields the average ranges illustrated in Figure 12B–D. The value ranges of the reaction time and conversion are wider (Figure 12C,D) for the clustering-supported approach, while the MSE value range is more restricted (Figure 12B). The findings indicate that the clustering approach allows for a better optimization of monomer conversion and reaction time, while the direct approach is better suited for the optimization of the MSEs.

Table 6 presents the execution time for both approaches for the test set **MMD**^test^ consisting of 810 target MMDs. For the clustering-supported approach, the optimization time depends slightly on the number of clusters. However, it is by a factor of ten smaller than that for the direct approach.

The rapidness of the MOO algorithm is especially important for the next step, when, in future genetic algorithms, together with ML models, it will be applied to extend the search space for the polymerization parameters, because Pareto optimization should be executed many times for each evolution stage of genetic algorithms. In this situation, clustering-supported Pareto optimization is preferable.

## 4. Conclusions and Outlook

This study bridges the gap between multi-objective optimization (MOO) and its application for the reverse engineering of polymerization processes. The proposed MOO models allow for the simulation-supported determination of an optimal recipe for a targeted molar mass distribution, taking multiple objectives, e.g., minimal reaction time, the maximal conversion of monomer, and the similarity of the found MMD compared to the target MMD into consideration. The proposed approach can be accelerated by additional clustering of the simulated MMDs. Moreover, it is possible to obtain a number of suitable candidates considering different weights of the selected objectives. A set of alternative optimal solutions is obtained for each specific combination of weights. First insights are provided on the formulation of a polymerization reverse engineering task as a MOO problem. The proposed MOO-based approach is a general method for solving reverse engineering problems; the objectives can be customized and extended. In future, this approach will be tested with other polymers. Moreover, the understanding gained in combination with previously proposed ML models [11] for polymerizations will be applied to facilitate and to speed up the discovery of optimal solutions with a limited number of kMC simulations. ML models can be used to set up the search space and to evaluate the candidate polymerization procedures rather than using kMC simulations. The search for candidate solutions will be performed with the help of genetic algorithms. Moreover, the consideration of more complex microstructural details, e.g., such as branching in the high-temperature polymerization of acrylate [36], may require the number of objectives in MOO to be increased. 

## Figures and Tables

**Table 1:** Optimization variables.

Variables	Description	Restrictions
*c*_m,0_	initial monomer concentration	*c*_m,0(min)_ ≤ *c*_m,0_ ≤ *c*_m,0(max)_
*c*_ini,0_	initial initiator concentration	*c*_ini,0(min)_ ≤ *c*_ini,0_ ≤ *c*_ini,0(max)_
*t*	reaction time	*t*_min_ ≤ *t* ≤ *t*_max_
**r** = [*c*_m,0_, *c*_ini,0_, *t*]	initial recipe	

**Table 2:** Optimization objective functions and their calculation on the basis of simulated data.

Objectives	Description
$\underset{r}{min}$ *f*_MSE_(**r**)=$\underset{r}{\min}\left[MSE({MMD}^{target},MMD(r))\right]$	minimal MSE, where *MMD*(**r**) is simulated
$\underset{r}{min}$ *f*_cm_(**r**)=$\underset{r}{\min}\left[1-f_{conv}(r)\right]=\underset{r}{\min}\frac{c_{m}\left(r\right)}{c_{m,0}}$	minimal relative monomer concentration
$\underset{r}{min}$ *f*_t_(**r**) = $\underset{r}{min}$ *t*	minimal reaction time (directly from **r**)

**Table 3:** The best recipes for each combination of objective weights (the focal weight is given in bold).

IDs of the Best Recipe		1	34	2		46	20	37
	*w*_i_	**Time Focus (A)**			*w*_i_	**Conversion Focus (B)**		
*c*_m,0_/mol∙L^−1^		2.0	2.21	2.0		2.21	2.43	2.43
*c*_ini,0_/mmol∙L^−1^		20	20	20		8.4	16.1	20
time/min	**0.9**	20	40	40	0.19	120	80	80
MSE/×10^−3^	0.05	1.63	1.37	0.08	0.01	1.72	1.58	1.81
conversion/%	0.05	25.9	46.6	45.9	**0.8**	71.3	69.6	73.7
**IDs of the Best Recipe**		**35**	**32**	**21**		**43**	**12**	**7**
	*w*_i_	**Equal Weights (C)**			*w*_i_	**MSE Focus (D)**		
c_m,0_/mol∙L^−1^		2.21	2.21	2.21		2.0	2.0	2.0
c_ini,0_/mmol∙L^−1^		20.0	10.5	13.0		12.4	10.0	1.5
time/min	**1/3**	60	80	80	0.01	180	200	160
MSE/×10^−5^	**1/3**	35	31	54	**0.9**	0.086	0.032	0.15
conversion/%	**1/3**	61.8	60.2	64.4	0.09	48.3	48.2	48.2

**Table 4:** Pareto front points **R**^par^ and their recipes and objective functions. Every highlighted point (bold letters) is the best result for the corresponding example objective weights.

Objective Weights	Cand. ID	c_m,0_/mol∙L^−1^	c_ini,0_/mmol∙L^−1^	Time/min	MSE/10^−3^	Conversion/%
	0	2.00	20.0	20	1.63	25.9
time focus: (*w*_cm_ = 0.2, *w*_t_ = 0.8)	**1**	**2.21**	**20.0**	**40**	**1.37**	**46.6**
	2	2.43	20.0	60	1.94	62.7
equal weights: (*w*_cm_ = 0.5, *w*_t_ = 0.5)	**3**	**2.43**	**20.0**	**80**	**1.81**	**73.7**
conversion focus: (*w*_cm_ = 0.8, *w*_t_ = 0.2)	**4**	**2.43**	**16.1**	**100**	**2.61**	**77.7**
	5	2.43	6.86	160	2.79	78.3
	6	2.43	5.54	180	2.83	78.5
	7	2.43	2.91	260	2.98	79.0

**Table 5:** Pareto optimal results with balanced weights for different numbers of clusters.

Number of Clusters		20	40	60
**Cand. ID/**		**3**	**2**	**1**
property	*w*_i_			
c_m,0_/mol∙L^−1^		3.50	3.07	2.86
c_ini,0_/mmol∙L^−1^		13.0	20.0	20.0
time/min	0.5	60.0	40.0	20.0
MSE/×10^−3^		3.04	0.32	0.03
conversion/%	0.5	58.2	49.2	27.0
score (*w*_t_ = 0.5, *w*_cm_ = 0.5)		0.50	0.39	0.50
score (*w*_MSE_ = 1/3, *w*_cm_ = 1/3, *w*_t_ = 1/3)		0.67	0.30	0.33

**Table 6:** Comparison of optimization time of direct and clustering-supported approaches.

Approach	Direct	Clustering-Supported Approach				
		**20 Clusters**	**40 Clusters**	**60 Clusters**	**80 Clusters**	**100 Clusters**
execution time, s	679	56	25	18	14	12

## Data Availability

The data presented in this study are openly available in Mendeley Data at https://doi.org/10.17632/hdrwmxgx27.1.
